# Affinity-Enhanced CTC-Capturing Hydrogel Microparticles Fabricated by Degassed Mold Lithography

**DOI:** 10.3390/jcm9020301

**Published:** 2020-01-21

**Authors:** Nak Jun Lee, Sejung Maeng, Hyeon Ung Kim, Yoon Ho Roh, Changhyun Hwang, Jongjin Kim, Ki-Tae Hwang, Ki Wan Bong

**Affiliations:** 1 Department of Chemical and Biological Engineering, Korea University, Seoul 02841, Korea; lemisily@gmail.com (N.J.L.); rlagus2009@gmail.com (H.U.K.); yoonho90@korea.ac.kr (Y.H.R.); changhwang77@gmail.com (C.H.); 2 Department of Surgery, Seoul Metropolitan Government Seoul National University Boramae Medical Center, Seoul 07061, Korea; skysj0718@gmail.com (S.M.); michael5@hanmail.net (J.K.)

**Keywords:** circulating tumor cell, cell capture, hydrogel microparticle, degassed mold lithography

## Abstract

Technologies for the detection and isolation of circulating tumor cells (CTCs) are essential in liquid biopsy, a minimally invasive technique for early diagnosis and medical intervention in cancer patients. A promising method for CTC capture, using an affinity-based approach, is the use of functionalized hydrogel microparticles (MP), which have the advantages of water-like reactivity, biologically compatible materials, and synergy with various analysis platforms. In this paper, we demonstrate the feasibility of CTC capture by hydrogel particles synthesized using a novel method called degassed mold lithography (DML). This technique increases the porosity and functionality of the MPs for effective conjugation with antibodies. Qualitative fluorescence analysis demonstrates that DML produces superior uniformity, integrity, and functionality of the MPs, as compared to conventional stop flow lithography (SFL). Analysis of the fluorescence intensity from porosity-controlled MPs by each reaction step of antibody conjugation elucidates that more antibodies are loaded when the particles are more porous. The feasibility of selective cell capture is demonstrated using breast cancer cell lines. In conclusion, using DML for the synthesis of porous MPs offers a powerful method for improving the cell affinity of the antibody-conjugated MPs.

## 1. Introduction

During the cancer metastatic process, malignant cells detach from the primary tumor, enter the blood stream, and relocate at a distal site. These circulating tumor cells (CTCs) serve as a tumor “fingerprint” in the bloodstream of cancer patients and may provide a minimally invasive target for early disease detection [1,2,3,4,5]. As most cancers remain asymptomatic at early stage and as advanced stages of cancer are associated with poor prognosis, the detection of CTCs may be an indicator of disease progression and an opportunity for early intervention treatments. While the validity of CTC detection, as a reliable and valuable biomarker of cancer metastatic progress, has been established by several large clinical studies [6], the extremely low number of CTCs in whole blood makes it difficult for clinical translations without specific techniques to detect and isolate CTCs [7]. For example, CTCs are rare in early breast cancer patients. Bidard et al. reported that approximately 50% of metastatic breast cancer patients exhibit no more than 5 CTC/7.5 mL of whole blood [8]. However, clinical studies of breast cancer patients have showed that CTC counts before chemotherapy intervention is a strong and independent prognostic indicator for distant metastasis free survival [9].

The potential clinical translation of CTC detection has motivated the innovation of several techniques for CTC isolation [10]. As a general approach, these technologies include the positive enrichment of CTC achieved through cancer cell-specific membrane antigens (e.g., EpCAM, CD45, MUC1, and so on) for capturing and imaging [11,12,13,14,15,16,17], or achieved through CTC physical characteristics (e.g., size base, dielectrophoresis, and so on) [18,19,20,21,22,23]. While each method has its own advantages, these platforms have limitations: size-based methods struggle with low recovery rates and processing challenges (e.g., sample clogging) [19,20], and fluorescence-activated cell-sorting and electrical approaches are not compatible with the scarcity of CTCs. Of the two general approaches, the most popular approach for CTC capture is affinity-based techniques [8,24,25,26,27,28,29], due to their high performance in CTC detection and isolation. They employ a wide range of specific monoclonal antibodies, lectins, and aptamers to monitor the various types of molecules on the cell surface. Using these cell surface markers, such techniques provide high cell selectivity and improved isolation purity by specific binding through the antigen–antibody interaction.

Using functionalized polyethylene glycol (PEG) hydrogel microparticles for CTC capture is a promising technique in the affinity-based approaches [30,31]. These microparticles are comprised of a network of cross-linked polyethylene glycol diacrylate (PEGDA), which is biologically inert, and provide a water-like environment. These networks are characterized by a large surface area to volume ratio and can contain over 90% water. The increased surface area enables ideal conditions for antibody immobilization, which is achieved through the binding of biotinylated antibodies to avidin protein, reducing the risk of steric effects [32,33,34]. Previously reported methods for the synthesis of these networked hydrogel particles have employed a platform called stop flow lithography (SFL) [27], in which UV light is used to polymerize the PEG hydrogels of various 2D extruded shapes inside a microfluidic channel. This platform enables control over the particle shape at high throughput synthesis efficiency. Chen et al. reported that a multi-armed shape hydrogel microparticle could provide a flexible structure with increased surface area for CTC capture [30].

For high capture efficiency, the particles should have a sufficient amount of antibodies for the selective identification of target cells. Antibody loading is maximized when the porous hydrogel structure is used as the anchoring platform [35]. To increase the porosity of a PEG hydrogel, a low content of cross-linking monomer is used with a high content of porogen in the prepolymer [36,37,38]. This synthesis condition is challenging to apply using the previously described SFL fabrication technique [30], as SFL has a short UV exposure time to facilitate its high throughput and microfluidic system stability, which produces particles with poor network integrity and, occasionally, even failure in the formation of particle synthesis. Increasing the UV intensity to offset the reduced exposure time produces particles that lack uniformity and compromises the SFL system with increases in microfluidic malfunctions (i.e., device clogging) [39,40]. Thus, the synthesis of porous particles suitable for antibody functionalization requires an alternative method, which is compatible with high-throughput processing and which yields uniform particles with a high surface to volume ratio.

In this work, we introduce a novel method for the fabrication of antibody-conjugated hydrogel microparticles called degassed mold lithography (DML) [41]. Unlike flow lithography, DML provides a wide range of UV conditions independent from the microfluidic conditions, eliminating the UV exposure limitations and fluid flow challenges. Due to its degassing effect, the oxygen inhibition effect is relieved, which allows the process to polymerize particles with high integrity and good uniformity. We produce particles using different prepolymer compositions to control the porosity. Using data from the analysis of fluorescence signals at each reaction step, we elucidate which factors critically affect the conjugation of antibodies. As proof of concept, optimized hydrogel particles were applied in a cell line assay to demonstrate that target cell capture can be achieved through selective antibody functionalization.

## 2. Experimental Section

### 2.1. Fabrication of the Micromold and Microfluidic Device

For the micromold used in degassed mold lithography (DML), the first step was the soft lithography of PDMS on a silicon master wafer, which had been patterned using an SU-8,25 photoresist (MicroChem) to form positive relief micro patterns of 25 µm height. The shape of the pattern was a square with 50 µm sides. PDMS (SYLGARD 184, Dow Corning), mixed at a 10:1 base to curing agent ratio, was poured over the master and cured for 4 h at 70 °C in an oven. Then, the cured PDMS was detached from the master. By the same process, PDMS blocks without pattern were prepared. For the microfluidic device used in stop flow lithography (SFL), a positive relief microchannel of 25 µm height was applied to PDMS. Then, the cured PDMS was detached from the master and perforated with 1.0 mm and 10.0 mm punches (Miltex) to make inlet and outlet reservoirs, respectively. A microscope slide was thinly coated with PDMS and partially cured at 70 °C for 30 min. Then, the perforated PDMS block and microscope slide were glued together, facing the adhesive surface, which is the negative relief side of PDMS block and surface of partially cured PDMS in a microscope. The two objects were placed in an oven and fully cured overnight.

### 2.2. DML Setup

The patterned PDMS micromold and PDMS block for covering were placed on a petri dish and degassed in a vacuum chamber at 0.1 atm. After about 30–60 min had elapsed, the cover block and the patterned block were removed from the vacuum chamber. Then, 30 µL of the prepolymer solution (Table 1) was added onto the cover block and the patterned mold was pressed down against the cover block, such that prepolymer solution on the cover filled the inside of the negative relief pattern. To eliminate unnecessary solution around the patterns, mold block complex was compressed at 1 atm for 20 s. Finally, UV with a wavelength of 365 nm was exposed to the mold blocks. A UV-LED lamp (SOLIS-365C, Thorlabs, Cambridge, UK) was controlled using designed LabVIEW code (National Instruments, TX, USA) and a DAQ board (Ni-DAQ, national instruments). The UV intensity was controlled by a power driver (DC20, Thorlabs).

### 2.3. SFL Setup

The stop flow lithography set-up involved using a UV-pressure system controlled by a LabView script. With the microfluidic device mounted on an inverted microscope (Axiovert 200, Zeiss), the prepolymer solution was loaded into the device inlet in a pipette tip reservoir. The reservoir was connected to a compressed air tank by a tygon tube, and the system was bridged by a pressure regulator (ITV0031-3BL, SMC pneumatics). A photomask, designed using AutoCAD and printed using a high-resolution printer (50,000 dpi), was inserted into the field-stop of the microscope. An LED lamp (M365LP1, Thorlabs) was used as the UV source for photo-polymerization [42,43]. In this work, the UV intensity was fixed at 300 mW/cm^2^, as measured through a 20× objective by a UV power meter (GT-510, Giltron).

### 2.4. Functionalization of Hydrogel Microparticle

The particle synthesis closely followed the previously reported protocol [30]. The functionalization process was identical for both particles synthesized by degassed mold lithography and stop flow lithography. Prepolymer compositions of the particles synthesized by DML and SFL are listed in Table 1. A prepolymer solution for the comparison between DML and SFL in Section 3.2 was used as described in Table 1. The prepolymer solution used consisted of Polyethylene glycol diacrylate (PEGDA700, Mn = 700 Da, Sigma-Aldrich), polyethylene glycol (PEG200; Mn = 200 Da, Sigma-Aldrich), acrylic acid (Sigma-Aldrich), and 2-Hydroxy-2-methyl propiophenone (photoinitiator, Sigma-Aldrich).

After the synthesis process, the particles were collected in a microtube and washed eight times. All particle functionalization steps required a particle concentration around 10,000 particles per mL of phosphate-buffered saline (PBS). N-(3-dimethylaminopropyl)-N’-ethylcarbodiimide hydrochloride (EDC, Sigma-Aldrich) and N-hydroxysuccinimide (NHS, Sigma-Aldrich) were dissolved separately in PBS for stock solutions. Then, the stock solutions were added to the rinsed particles to achieve final concentrations of 3.0 mg/mL each. The particle solution was vortexed for 1 min and treated by the ultrasonic batch for 1 min to prevent aggregation. Then, a tube containing the solution was placed on an incubating shaker (1500 rpm) at 25 °C for 30 min. After activation of the NHS ester, particles were rinsed six times in PBST—a combination of 1 × PBS (without calcium and magnesium, Corning) and 0.05% (*v*/*v*) tween 20 (Sigma-Aldrich)—by centrifugation. Additional vortexing and sonication prevented the aggregation of particles in solution. After washing, NeutrAvidin (ThermoFisher) stock solution diluted with ultrapure water and 10% (*v*/*v*) glycerol (Sigma-Aldrich) was added to the particles to achieve a final protein concentration of 1.0 mg/mL and the solution was placed on an incubating shaker (1500 rpm) at 25 °C for 3 h. The particles were rinsed six times in PBST and biotinylated anti-EpCAM (Invitrogen) was added to achieve a concentration of 0.001 mg/mL. Particles were rinsed six times and stored at 4 °C.

### 2.5. Fluorescence Analysis

All steps for conjugating fluorescent dyes were identical to the functionalization of the hydrogel microparticles. Alexa fluor 488 cadaverine (ThermoFisher), biotin-PEG-FITC (Mn = 1000 Da, Nanocs), and Goat anti-Mouse IgG recombinant secondary antibody conjugated with Alexa Fluor 488 (ThermoFisher) were applied to carboxyl group, NeutrAvidin, and anti-EpCAM for quantification, respectively.

### 2.6. Cell Culture

Three human breast cancer cell lines, including high-EpCAM-expressing MCF-7, meddle-EpCAM-expressing SK-BR3, and non-expressing EpCAM MDA-MB231, were obtained from the American Type Culture Collection (ATCC, Rockvile, MD, USA). MCF-7 cells were propagated in RPMI supplemented with 10% fetal bovine serum (MPbio) and 1% antibiotic-antimycotic (Gibco). MDA-MB231 cells were propagated in Dulbecco’s modified eagle medium (DMEM) supplemented with 10% FBS and 1% Antibiotic. SK-BR3 cells were propagated in McCoy’s 5A supplemented with 10% FBS and 1% Antibiotic. Cell lines were maintained at 37 °C in a humidified atmosphere with 5% CO_2_. For cell-particle experiments, cells were subsequently trypsinized using TrypLE Express without Phenol Red (Thermo), and then resuspended in 0.1% BSA/PBS buffer at a concentration of 3 × 10^4^ cells/mL.

### 2.7. Cell Affinity Test

Particles (500 particles) and cells (30,000 cells) were incubated together for 2 h at room temperature, under gentle agitation. Total solution volumes were 1 mL. Before imaging, solutions were vortexed gently to remove non-adherent cells from particles using a 40 μm cell strainer (SPL). Finally, the cell-particles were observed by inverted microscopy (Leica DMIL) and cultured 12-well at 37 °C in a humidified atmosphere with 5% CO_2_. All assays were done in triplicate, and the data were expressed as mean ± SD.

### 2.8. Image Analysis

Monochrome fluorescence images of the particles were taken on an inverted fluorescence microscope (Axiovert 200, Zeiss, Germany) connected with a scientific complementary metal-oxide-semiconductor (CMOS) camera (Prime, Photometrics, USA) and a fluorescence light source (Illuminator HXP 120V, Zeiss, Germany). For detecting fluorescence signals from Alexa fluor 488 cadaverine, biotin-PEG-FITC, and Alexa fluor 488 conjugated 2nd antibody, the light was filtered using a green fluorescence filter set (λex/λem = 492/517 nm) [44,45]. The images were taken using 20× objective with a camera exposure time of 100 ms. The UV intensity of the fluorescence light source was fixed at 1100 mW/cm. The image was saved in TIFF format for the image processing program ImageJ (NIH). Statistical significance between SFL and DML was assessed by Student’s t test using the SPSS software version 25 (IBM, Armonk, NY, USA).

## 3. Results

### 3.1. Functionalized Hydrogel Microparticle Synthesis via DML

**Figure 1:**
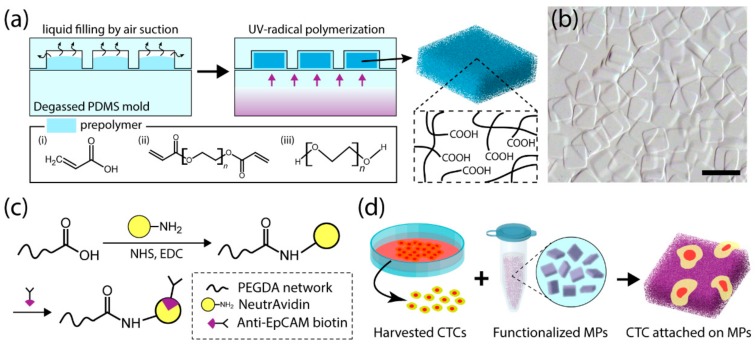
Schematic view of particle synthesis and cell test: (**a**) Particle synthesis process called degassed mold lithography (DML). Through UV-induced radical polymerization with the prepolymers (i) acrylic acid, (ii) polyethylene glycol diacrylate (PEGDA), and (iii) polyethylene glycol (PEG), hydrogel microparticles containing carboxyl groups are synthesized. (**b**) Bright-field image of polymerized hydrogel microparticle. Scale bar is 200 µm. (**c**) Carbodiimide cross-link chemistry is used to conjugate anti-EpCAM to carboxyl groups. Carboxyl groups in the particles react to primary amines in NeutrAvidin with the help of N-hydroxysuccinimide (NHS) and N-(3-dimethylaminopropyl)-N’-ethylcarbodiimide hydrochloride (EDC). Using the interaction between avidin protein and biotin, biotin-conjugated anti-EpCAM is applied. (**d**) Functionalized hydrogel microparticles capture circulating tumor cells (CTCs) by antigen–antibody interaction.

To produce functionalized hydrogel microparticles, we adopted DML techniques for the synthesis of porous hydrogel microparticles. For functionality, acrylic acids were added to the PEGDA-based prepolymers to form carboxyl groups. The PEG in the prepolymers served as a porogen to facilitate particle porosity. The prepolymer solution was loaded into the PDMS mold blocks and degassed within the vacuum chamber. Once degassed and removed from the chamber, the PDMS block absorbed the surrounding air due to the pressure differential generated across the interior and exterior of the block. As a result, the degassed mold block drew the prepolymer solution into the mold pattern. Covered by the plain PDMS mold, the solution was trapped inside the hollow regions of the mold (Figure 1a). Once the prepolymer solution was loaded into the mold, the PDMS block was exposed to ultraviolet rays to initiate UV-radical polymerization. For this study, micromolds patterned with square shape were used to demonstrate proof of concept. Figure 1b shows a bright-field image of the particles synthesized by DML.

For functionalization, carbodiimide cross-link chemistry was used to conjugate antibodies through carboxyl groups. Traditional NHS and EDC chemistry was applied to covalently attach NeutrAvidin protein to the carboxyl groups. Consequently, using a biotinylated antibody for the avidin–biotin reaction, anti-EpCAM biotin was conjugated to the NeutrAvidin protein (Figure 1c). Finally, in cell line evaluations, functionalized particles (MPs in Figure 1d) were mixed with breast cancer cells to evaluate the selectivity and efficiency of cell capture.

### 3.2. Comparison of the Particle Properties between DML and SFL

**Figure 2:**
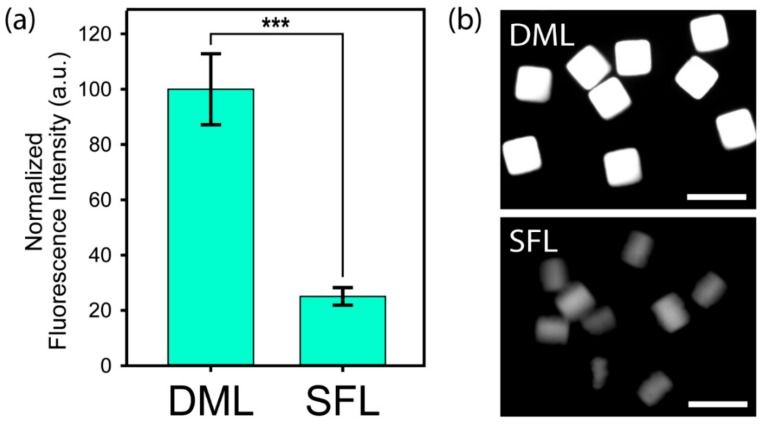
Performance comparison between DML and SFL: (**a**) Normalized fluorescence intensity of Alexa fluor 488 cadaverine conjugated directly to carboxyl groups. Prepolymer composition of 2.5% (*v*/*v*) PEGDA 700, 62.5% (*v*/*v*) PEG, 30% (*v*/*v*) acrylic acid, and 5% (*v*/*v*) PI was used. *** *p* < 0.001 compared to the SFL. (**b**) Fluorescence images of synthesized particles through DML (**top**) and SFL (**bottom**). Scale bars are 200 µm.

We quantitatively compared the performance of the DML and SFL methods by synthesizing particles using the same prepolymer conditions (PEGDA 700 at 2.5%, PEG 200 at 62.5%, Acrylic acid at 30.0%, and photoinitiator at 5.0%). Particles were synthesized under the same UV conditions for both methods. Alexa fluor 488 cadaverine, a fluorescence marker which is reactive to the carboxylic acid moiety, was used for quantifying functionality (Figure 2a). The results shown in Figure 2b revealed that, under low monomer concentration in the prepolymer, degassed mold lithography produced particles with good uniformity and functionality, but SFL produced particles with distorted shapes and poor functionality. This result supports that the critical percentage of PEGDA required to initiate gelation through SFL is 2%, which is close to the value of 2.5% PEGDA used in the prepolymer composition [46,47]. Therefore, it has been demonstrated that DML has a superior ability to SFL in synthesizing particles from a prepolymer solution consisting of a cross-linker at low concentration.

To produce particles through DML in an optimal manner for the experiments, we performed a reproducibility test with modulation of UV intensity and antibody reaction concentration. The particles fabricated by DML displayed good reproducibility in size (intra c.v. < 5%, inter c.v. < 5%) and functionality (intra c.v. < 15%, inter c.v. < 10%) (see Figures S1 and S2). The UV conditions of particle synthesis and antibody reaction were also optimized for the experiment (see Figures S3 and S4).

### 3.3. Qualitative Analysis of Surface Carboxyl Group

The functionalization of anti-EpCAM to the hydrogel particles is dependent on the availability of the carboxyl group, as this functional group is the target of the NeutrAvidin conjugation. To identify the optimal prepolymer conditions for particle functionalization, the presence of carboxyl group was evaluated using the formulation presented in Table 1. Alexa fluor 488 cadaverine was chosen for quantification of the carboxyl group, due to its low molecular weight (~600 Da) compared to NeutrAvidin (~60,000 Da), which is favorable for diffusing deep inside the hydrogel network.

**Figure 3:**
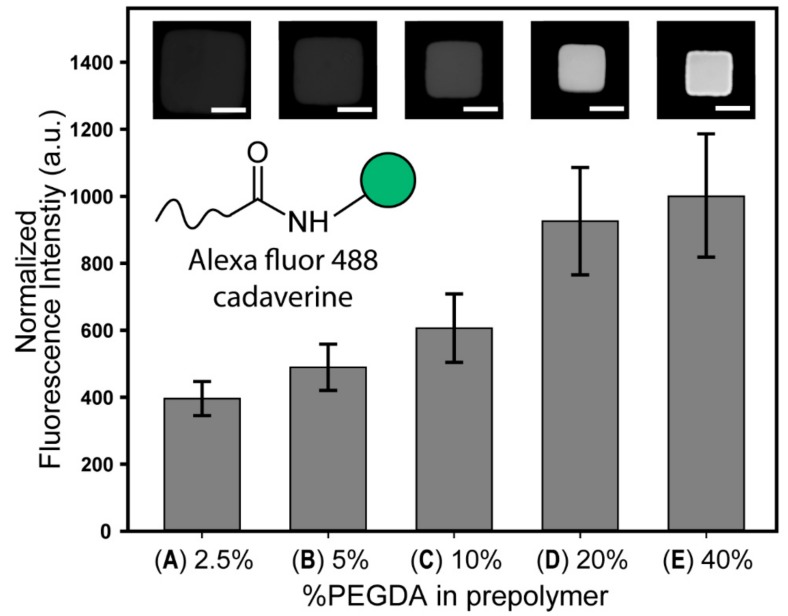
Characterization of fluorescence intensity obtained from carboxyl groups in the particles. Carboxyl groups are conjugated with Alexa fluor 488 cadaverine by carbodiimide chemistry. Fluorescence intensity is normalized on the basis of group E (40% PEGDA in prepolymer; Table 1). Each bar and error bar represent the average signal and standard deviation of 10–15 particles. Each image in the graph corresponds to the bar directly below. All the images are shown in same calibration profile of brightness and contrast. Scale bars are 50 µm.

We estimated that a higher concentration of cross-linker in the prepolymer solution could increase the carboxyl group in the polymer network of the synthesized particles. To demonstrate our hypothesis, we synthesized particles with different ratios of cross-linkers in the prepolymer (Table 1). As the ratio of cross-linking monomers (%PEGDA) increased, the density of the hydrogel structure increased, which means more carboxyl groups were present (Figure 3). The size of the particles was bigger with low cross-linking monomer concentration in the prepolymer, due to swelling of the polymer network caused by negatively charged carboxyl groups. In order to compensate for the effect caused by hydrogel swelling, the fluorescence intensity value was calibrated by the value divided by the ratio of particles to the size of micromold.

### 3.4. Qualitative Analysis of NeutrAvidin and Anti-EpCAM Functionalization

**Figure 4:**
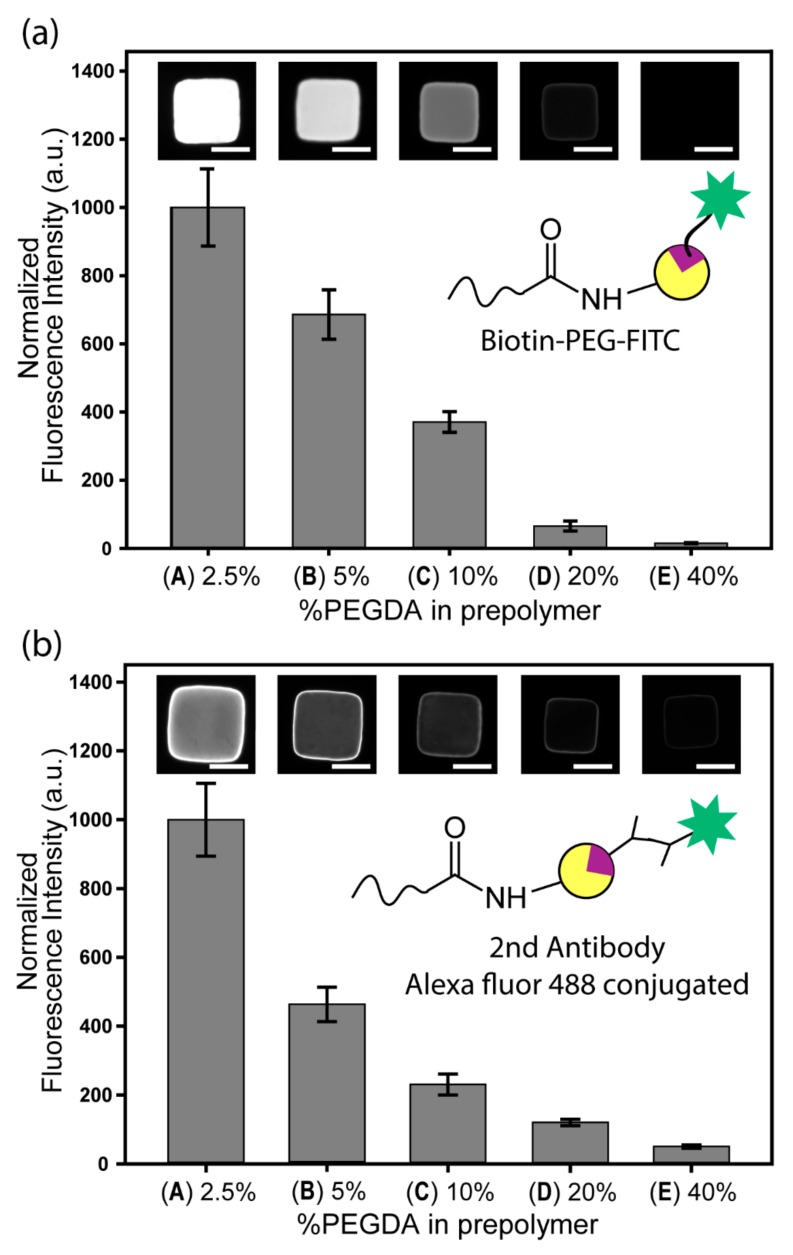
Characterization of fluorescence intensity obtained from NeutrAvidin proteins and antibodies in the particles: (**a**) NeutrAvidin proteins are conjugated with biotin–PEG–FITC by avidin–biotin interaction. Fluorescence intensity is normalized on the basis of group A (2.5% PEGDA in prepolymer). (**b**) Secondary fluorescence antibodies conjugated with Alexa fluor 488 are attached to the biotinylated EpCAM antibodies for quantification. Each data bar and error bar represent the average signal and standard deviation of 10–15 particles. Each image in the graph corresponds to the bar directly below. All the images are shown in same calibration profile of brightness and contrast. Scale bars are 50 µm.

Surprisingly, an increase in carboxyl groups did not translate to an increase in the amount of NeutrAvidin attachment (Figure 4a). Biotin–PEG–FITC (~1000 Da) was chosen as a fluorescence marker for the quantification of conjugated NeutrAvidin protein to the particle. Fluorescence analysis revealed that the presence of NeutrAvidin proteins decreased when the PEGDA ratio increased, suggesting that particles that were less porous exhibited lower NeutrAvidin content—a result that was the opposite of the carboxyl groups evaluation (Section 3.3).

In the case of anti-EpCAM antibody conjugation, this tendency was more pronounced (Figure 4b). Compared to Figure 4a, the fluorescence intensity increased steeply with a decrease of PEGDA content. As the molecular weight of anti-EpCAM (~150,000 Da) is larger than that of NeutrAvidin (~60,000 Da), it was much more difficult for antibodies to penetrate into the particle than NeutrAvidin. We hypothesized that the EpCAM antibodies would have limited access to the interior of the particles because of the previously bounded NeutrAvidin’s physical presence introducing steric hindrance and reduction of diffusion. Thus, the process of conjugating antibodies was affected by the particle’s porosity. These differences between antibody and protein could also be confirmed by comparing fluorescence intensities on the edge side and internal side of the particle; the contrast between the edge side and internal side was greater in the case of anti-EpCAM than in NeutrAvidin, which meant diffusion of anti-EpCAM in the reaction was much more difficult than that of the NeutrAvidin protein (Figure S5).

### 3.5. Cell Affinity Test

**Figure 5:**
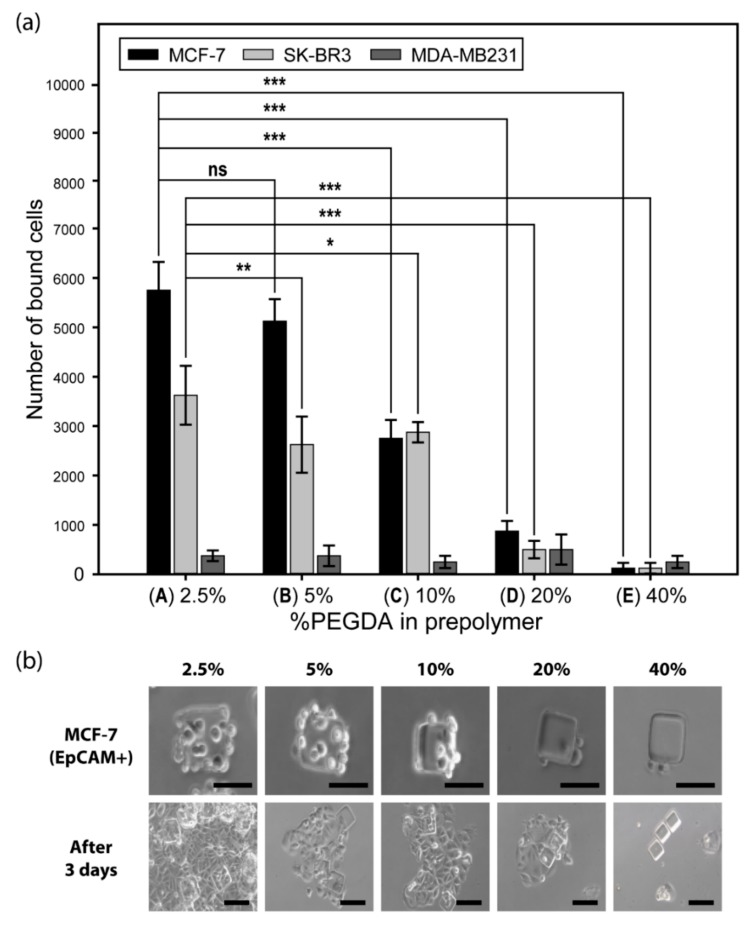
Cell capture by different prepolymer composition: (**a**) EpCAM expressing cells (MCF-7, SK-BR3) are captured by anti-EpCAM conjugated particles, while non-EpCAM expressing cells (MDA-MB231) do not respond. Each data bar and error bar represent the average signal and standard deviation of >10 particles (ns: *p* > 0.05, *: *p* < 0.05, **: *p* < 0.01, ***: *p* < 0.001, by Student’s *t*-test with Bonferroni correction). (**b**) EpCAM expressing cells (MCF-7) are successfully cultured on the particles. All scale bars are 100 µm.

An optimization study revealed that particles synthesized with a prepolymer condition of lower PEGDA concentration yielded higher surface functionalization of the EpCAM antibody. The performance of these particles to selectively capture EpCAM positive cell lines was evaluated. Three breast cancer cell lines were used as proof of concept: MCF-7, SK-BR3, and MDA-MB231. MCF-7 and SK-BR3 express surface EpCAM antigen. MDA-MB231 does not express EpCAM and serves as a control for antibody specificity. As Figure 5a indicates, functionalized hydrogel microparticles effectively captured only EpCAM expressing cells. However, the particles did not capture MDA-MB231, further supporting enrichment specificity. In addition, non-functionalized particles did not exhibit affinity toward all types of cells (no data shown).

Figure 5b demonstrates that captured EpCAM positive cells were successfully cultured with particles attached to them. The culture condition did not require the separation or post-treatment of the cells from the particles, due to the biocompatibility of PEG-based hydrogels [48].

Finally, we compared the cell affinity of the porosity-tuned particles synthesized with DML with particles synthesized using SFL, where the UV conditions and particle shapes for particle synthesis were kept constant. Comparison of the results showed a higher cell affinity of the particles synthesized through DML (Figure S6).

## 4. Discussion

In this study, we demonstrated the feasibility of selectively capturing cancer cells by hydrogel particles synthesized using our DML technique. The DML technique enables high throughput synthesis of the described hydrogel particles without the limitations of short UV exposure and microfluidic challenges associated with conventional approaches, such as SFL. The DML technique provides a wide range of UV exposure times, owing to the stationary condition of the prepolymer inside the degassed mold, and can produce a massive amount of particles with a single UV exposure. However, in SFL, long UV exposure leads to decrease in the synthesized particles per production cycle and can cause stability issues, such as clogging inside the microchannel. Low UV exposure is technically insufficient for the synthesis of hydrogel particles using prepolymers containing low cross-linker content. The presented DML technique enables the use of a low prepolymer condition for particle synthesis. Therefore, porous particles with high uniformity and integrity can be produced by DML at high throughput.

In DML, we optimized the prepolymer solution condition to maximize the functionalization of the CTC-specific antibody (EpCAM). While it is logical to speculate that a higher concentration of carboxyl groups would yield higher NeutrAvidin functionalization, qualitative assessment of NeutrAvidin binding demonstrated the opposite: particles synthesized with a lower number of carboxyl groups produced better NeutrAvidin functionalization. This was because steric hindrance limited the number of protein attachments. The NeutrAvidin proteins were not bound to all of the available carboxyl groups, due to their large molecular weight. The antibody conjugation level was more affected by the porosity in the particle, rather than the density of carboxyl groups.

Hydrogel particles synthesized using optimized prepolymer conditions exhibited sufficient conjugation of capture antibodies for the selective capture of cells. A correlation between the number of captured cells and PEGDA percentages in the prepolymer composition was confirmed: porous particles containing relatively more antibodies tended to have higher cell-capturing performance. We demonstrated that only the cells expressing surface EpCAM antigen were captured by the particles loaded with the anti-EpCAM antibody. Cells that did not express this marker were not isolated. Selective cell capture could be more tailored through antibody selection.

We believe that our technique could be useful for the specific enrichment of cancer cells for early diagnostic application. As a proof of concept, we demonstrated the feasibility of capturing breast cancer cell lines from solution through EpCAM functionalized hydrogel microparticles synthesized using our DML technique. Furthermore, the successful culture of these captured cells implies that DML particles does not hinder cell proliferation. This result indicates not only the biocompatibility of the particles, but also the potential of this technique, enabling the capture and culture of enriched cells in an integrated one-step process for patient-specific treatment.

## Figures and Tables

**Table 1:** Prepolymer composition of the five tested groups.

Group	Composition			
	PEGDA 700	PEG 200	Acrylic Acid	PI
A	2.5%	62.5%	30.0%	5.0%
B	5.0%	60.0%	30.0%	5.0%
C	10.0%	55.0%	30.0%	5.0%
D	20.0%	45.0%	30.0%	5.0%
E	40.0%	25.0%	30.0%	5.0%

PEGDA: Polyethylene glycol diacrylate; PEG: Polyethylene glycol; PI: Photoinitiator.

## Supplementary Materials

The following are available online at https://www.mdpi.com/2077-0383/9/2/301/s1, Figure S1: Fluorescent intensity of Alexa 488 cadaverine according to UV intensity, Figure S2: Reproducibility test of particle size, Figure S3: Reproducibility test of functionality through carboxyl group, Figure S4: Fluorescent intensity of Alexa 488 conjugated 2nd antibody according to antibody concentration, Figure S5: Comparison of contrast in the fluorescence intensity between data from anti-EpCAM and NeutrAvidin protein, Figure S6: Cell affinity comparison between particles synthesized by DML and SFL.
