# Supplementary material for: Affinity-Enhanced CTC-Capturing Hydrogel Microparticles Fabricated by Degassed Mold Lithography
Source: J Clin Med. 2020 Jan 21;9(2):301. doi: 10.3390/jcm9020301 (PMC7073783; doi:10.3390/jcm9020301)
Supplement: Supplementary file 1 [file jcm-09-00301-s001.pdf]

Supporting information for

Affinity-enhanced CTC-Capturing Hydrogel Microparticles fabricated by Degassed Mold Lithography

Nak Jun Lee [1,†], Sejung Maeng [2,†], Hyeon Ung Kim [1], Yoon Ho Roh [1], Changhyun Hwang [1], Jongjin Kim [2], Ki-Tae Hwang [2,*], and Ki Wan Bong [1,*]

1. Department of Chemical and Biological Engineering, College of Engineering, Korea University, Seoul 02841, Republic of Korea
2. Department of Surgery, Seoul Metropolitan Government Seoul National University Boramae Medical Center, Seoul 07061, Republic of Korea

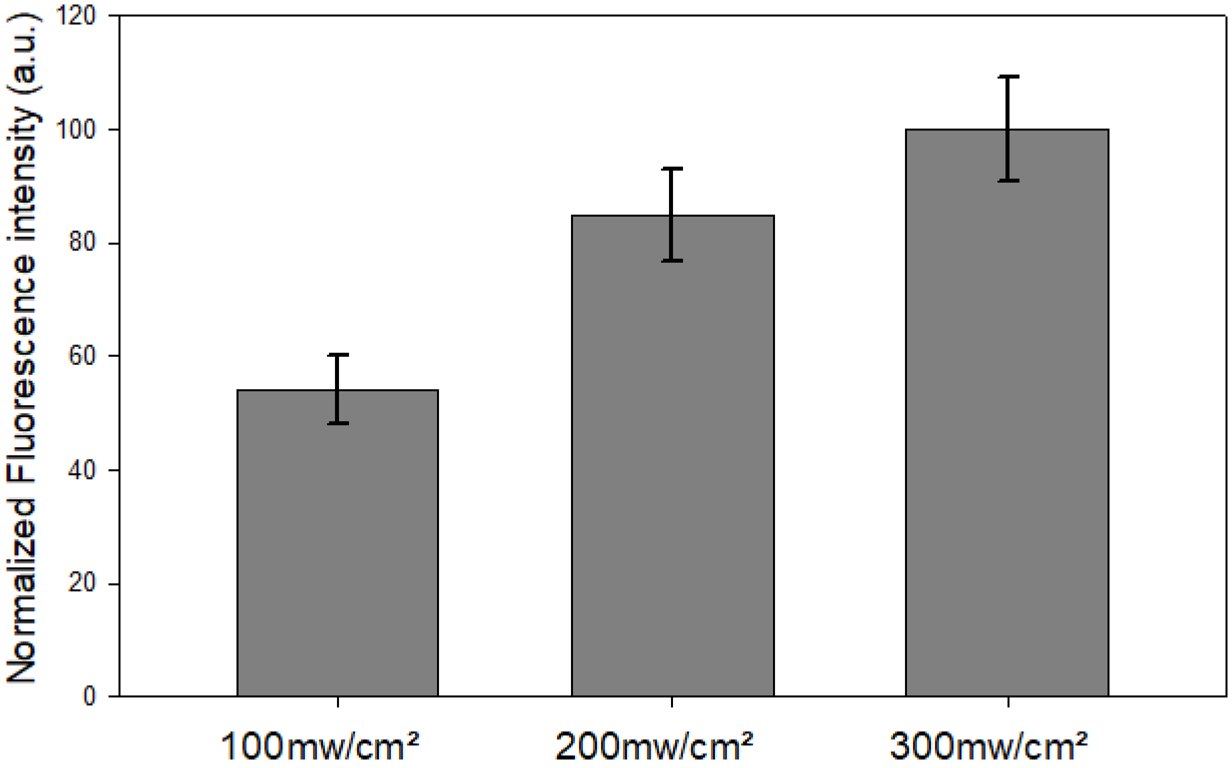

Figure S1 Fluorescent intensity of Alexa 488 cadaverine according to UV intensity.

UV exposure time is fixed to 300 ms. All particles are identical in three cases. Each bar data and error bar represent the average signal and standard deviation of 10~15 particles.

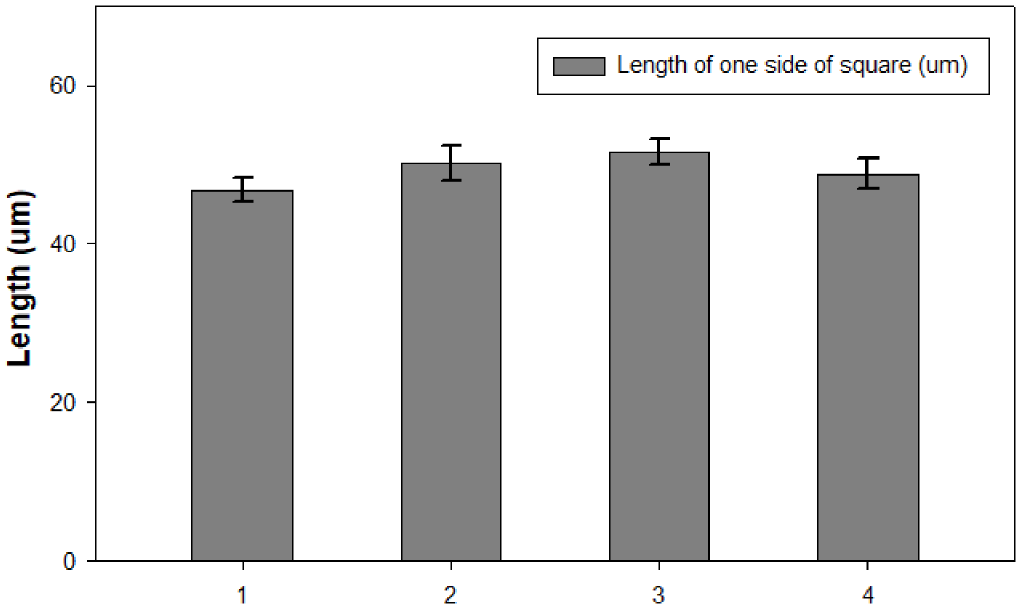

Figure S2 Reproducibility test of particle size.

Each bar data and error bar represent the average signal and standard deviation of 10~15 particles. We performed four sets of DML and each set represents the experiment done by single mold.

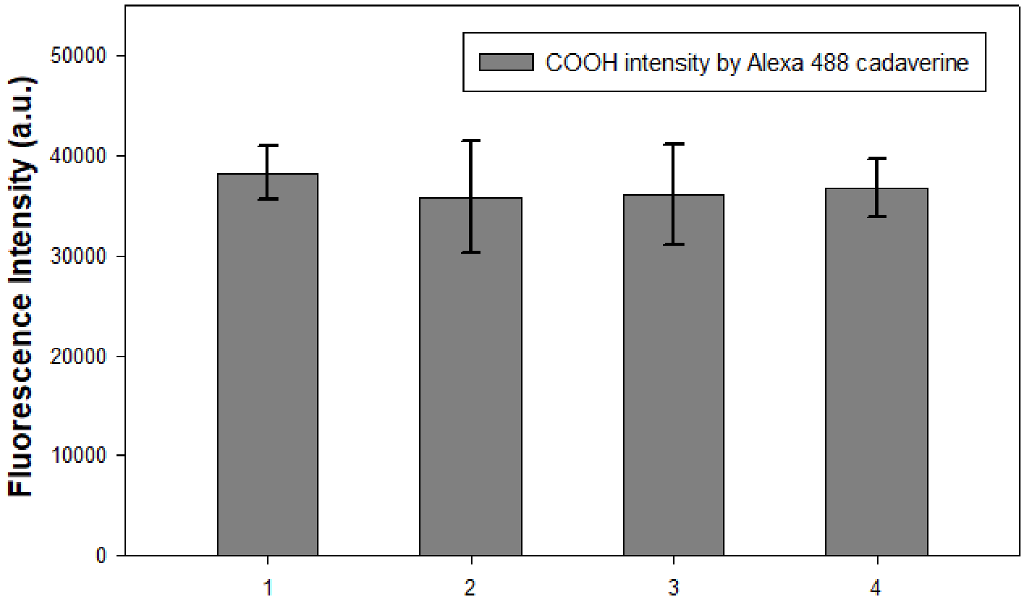

Figure S3 Reproducibility test of functionality through carboxyl group.

Each bar data and error bar represent the average signal and standard deviation of 10~15 particles. We performed 4 sets of DML and each set represents the experiment done by single mold.

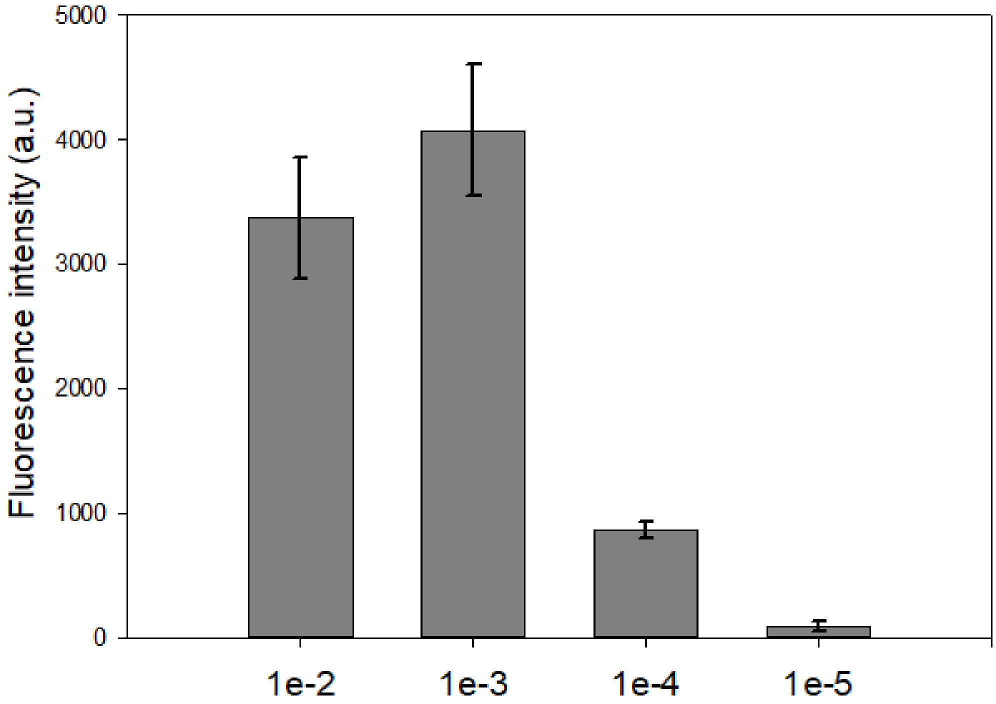

Figure S4 Fluorescent intensity of Alexa 488 conjugated 2nd antibody according to antibody concentration.

Unit of the value in x-axis is mg/mL. Each bar data and error bar represent the average signal and standard deviation of 10~15 particles.

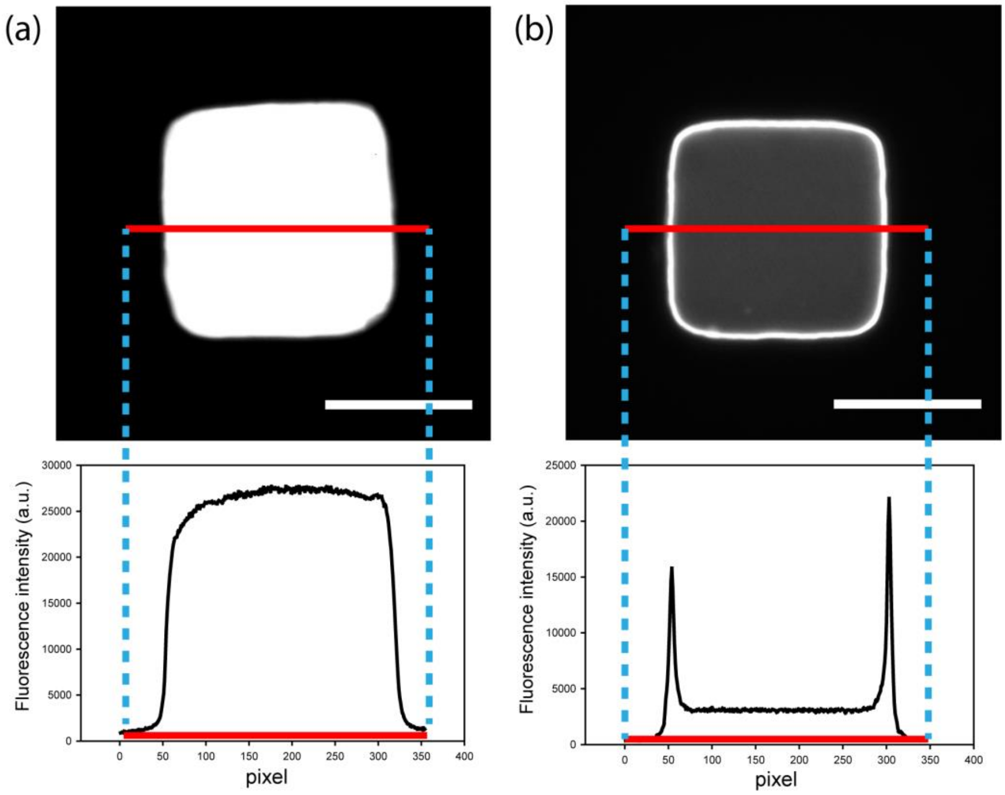

Figure S5 Comparison of contrast in the fluorescence intensity between data from anti-EpCAM and NeutrAvidin protein

Red line indicates the measured point of the fluorescent signal. Signal is obtained by ImageJ. The scale bar is 50 µm.

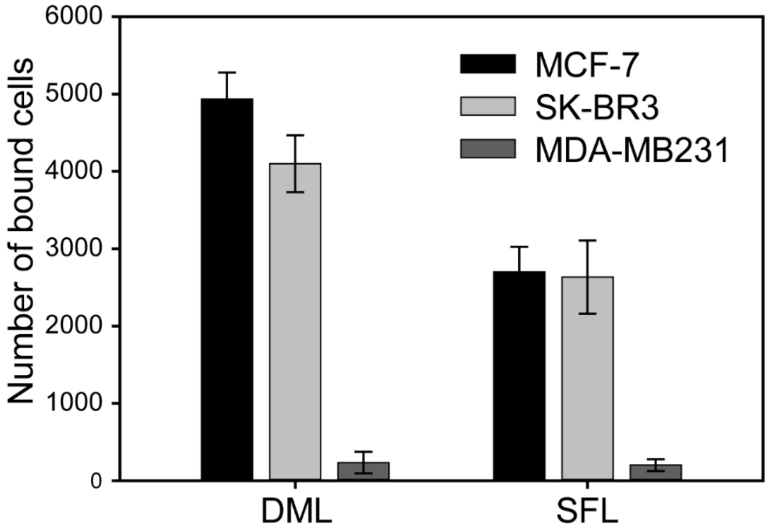

Figure S6 Cell affinity comparison between particles synthesized by DML and SFL

Each bar data and error bar represent the average signal and standard deviation of >10 particles. For SFL, a prepolymer solution used to synthesize particles consisted of 30% (v/v) PEGDA 700, 35% (v/v) PEG 200, 30% (v/v) acrylic acid, and 5% (v/v) PI.
